# Perioperative airway management for aortic valve replacement in an adult with mucopolysaccharidosis type II (Hunter syndrome)

**DOI:** 10.1186/s40981-018-0162-5

**Published:** 2018-03-05

**Authors:** Kazuchika Suzuki, Hiroaki Sakai, Kenji Takahashi

**Affiliations:** 1 Department of Cardiovascular Surgery, Fujieda Municipal General Hospital, 4-1-11 Surugadai, Fujieda, Shizuoka Japan; 2 Department of Anesthesia, Fujieda Municipal General Hospital, 4-1-11 Surugadai, Fujieda, Shizuoka Japan

**Keywords:** Hunter syndrome, Adult, Airway management

## Abstract

We herein report anesthetic management during aortic valve replacement for aortic valve regurgitation in a patient with adult mucopolysaccharidosis type II (MPS type 2) (Hunter syndrome). This disorder is rare and related to the accumulation of a mucopolysaccharide in lysosomes. It affects various organs, including the airways, heart, and central nerves. In children with MPS type 2, the risk of airway obstruction during anesthesia/sedation is high, and the degree of difficulty increases with aging. The patient described herein was a 33-year-old male without mental retardation. Before surgery, trismus, megaloglossia, and the disturbance of cervical vertebral excursion were noted, suggesting difficulties with ventilation/intubation. Anesthesia was induced under sedation/spontaneous respiration. A laryngeal deployment was conducted using a video laryngoscope; however, the Cormack grade was III. Nasotracheal fiber intubation was performed, and airway obstruction occurred. A muscle relaxant was administered, facilitating ventilation. However, subglottic stenosis, which was not detected before the surgery, made the tracheal tube insertion difficult. Aortic valve replacement was performed without complications. A detailed postoperative examination of the airways revealed oropharyngeal soft tissue outgrowth, narrowing of the upper airway, subglottic stenosis, and displacement/circumflex of the airway axis. Either awake intubation or rapid induction can be selected for this patient; however, either way have risks of airway obstruction. It is important that strategies under light anesthesia or incomplete neuromuscular blockade should be avoided for such our patient as suggested in the JSA airway management guidelines. A preoperative multidisciplinary airway assessment and simulation are important.

## Background

We performed anesthetic management during aortic valve replacement for aortic valve regurgitation in a patient with adult mucopolysaccharidosis type II (MPS type 2) (Hunter syndrome). MPS type 2 is a sex-linked recessive disorder and is related to the accumulation of a mucopolysaccharide, glycosaminoglycan (GAG), in lysosomes, which results from the disturbance of mucopolysaccharide metabolism by intracellular lysosomes. It affects various organs, including the airways, heart, and central nerves. In children with MPS type 2, the risk of airway obstruction during anesthesia/sedation is high, and the degree of difficulty increases with aging [1, 2]. Furthermore, this disorder may cause heart valve disease, cardiomyopathy, coronary artery stenosis, and disturbance of the conduction system [2, 3]. Few studies have reported anesthetic management during cardiac surgery in adults with MPS type 2. We herein describe anesthetic management in a patient with adult MPS type 2 for whom general anesthesia was induced in consideration of difficulties with intubation, and airway management was more challenging than expected.

## Case presentation

The patient was a 32-year-old male with MPS type 2. His height and body weight were 134 cm and 47 kg, respectively. When he was an elementary school student, he underwent surgery for an inguinal hernia. Mental retardation was not indicated. He (first-born child) had two brothers. They also had the same disease and underwent aortic valve replacement when they were elementary school students. At the age of 13 years, the patient was diagnosed with aortic valve regurgitation. Since 19 years of age, he had been followed up in the Department of Cardiology of our hospital. Aortic valve replacement was performed due to an increase in the regurgitation volume. There were no heart failure-related symptoms, and the NYHA grade was evaluated as I. Preoperative electrocardiography showed a sinus rhythm, and echocardiography revealed moderate aortic valve regurgitation, an end-diastolic left ventricular diameter of 56 mm and left ventricular ejection fraction of 51%. Coronary angiography did not show any significant stenosis.

**Fig. 1:**
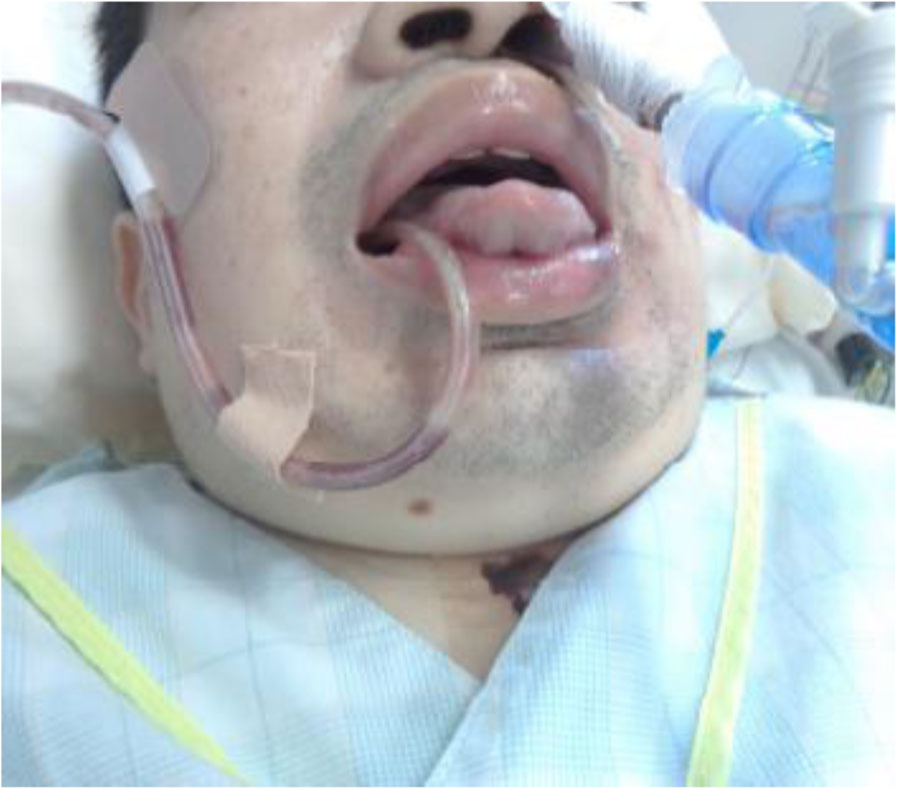
At first postoperative day, after confirming mouth opening and tongue motility, extubation was conducted. Patient revealed trismus, brevicollis, and megaloglossia

**Fig. 2:**
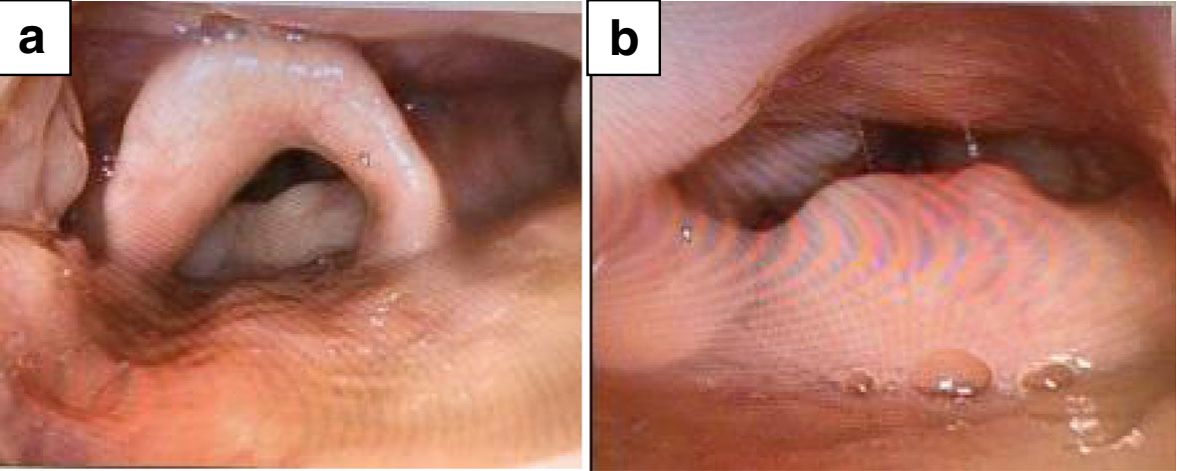
After surgery, the larynx was examined using an endoscope. **a** Soft tissue outgrowth at the site of the fissure. **b** Difficulties were associated with visually recognizing the glottis and subglottic area

**Fig. 3:**
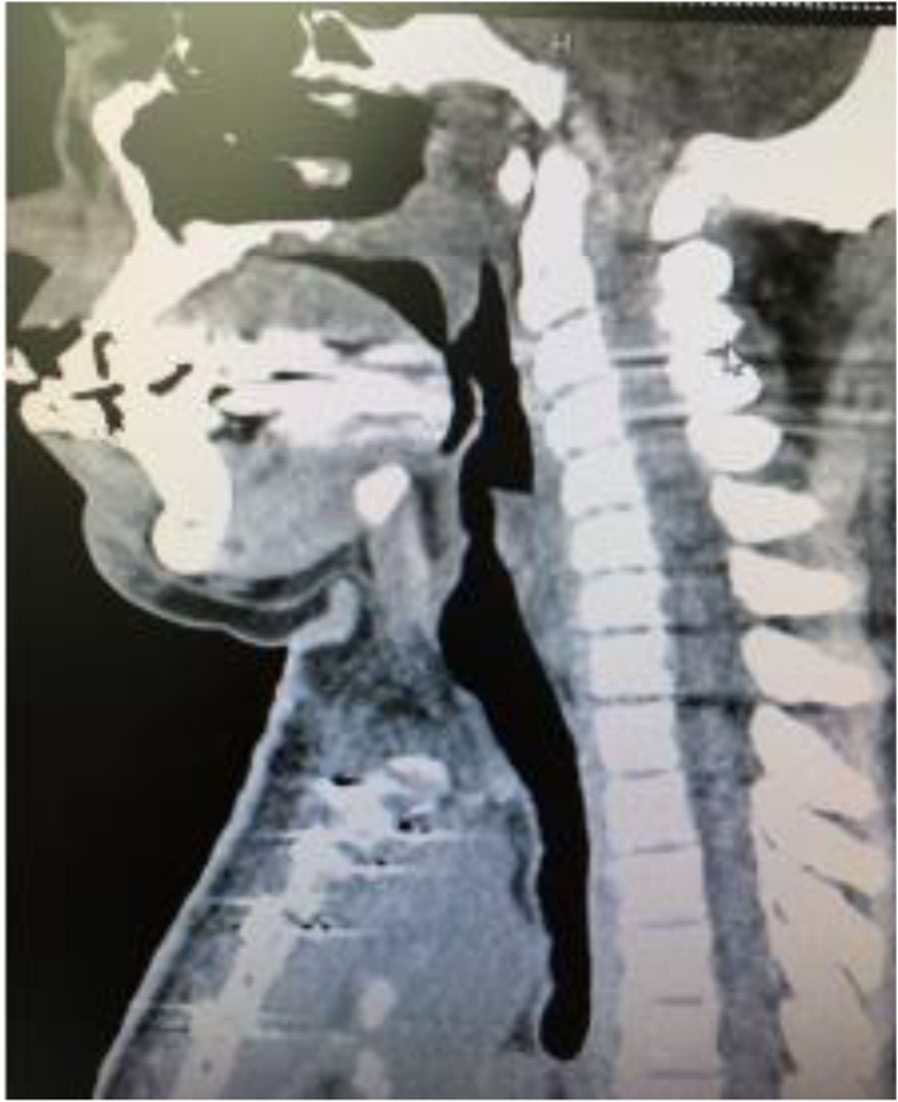
Cervical CT revealed that cervical vertebrae comprised a straight neck, glottis were present at the level of the sixth cervical vertebra, and the airway patency was maintained

**Fig. 4:**
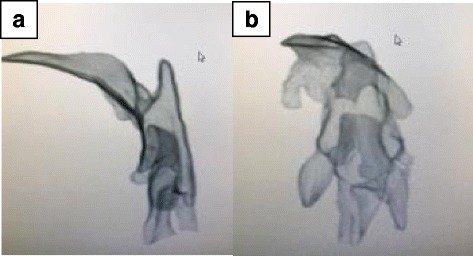
Postoperative 3D CT image of the upper airway. **a** Lateral view of the upper airway on 3D CT. **b** On a dorsal view, circumflex of the upper airway was noted

A preoperative examination showed trismus (Mallampati grade IV), brevicollis, posterior cervical flexure disturbance, and megaloglossia. Coronal sections on magnetic resonance imaging (MRI) indicated similar findings, suggesting that airway management is difficult. Preanesthetic medication was not performed. After the patient was admitted to the operating room, a percutaneous oxygen saturation monitor, electrocardiograph, non-invasive blood pressure monitor, Bispectral Index monitor, and near infrared ray brain tissue oxygen saturation monitor were attached. In addition to the intravenous administration of fentanyl at a total dosage of 200 μg and midazolam at 2.5 mg, propofol at 1.0 mg/kg/h and remifentanil at 2.0 μg /kg/h were continuously administered under oxygen administration. The Richmond Agitation Scale Score (RASS) ranged between − 1 and − 2. Under spontaneous respiration, a 22G needle was inserted into the right radial artery, and continuous arterial pressure monitoring was started. The sedation level reached the degree of mouth opening in response to a strong call, and 8% lidocaine spray was sprayed into the oral cavity for local anesthesia. A McGrath® X blade™ was inserted to spread the larynx; however, difficulties were associated with inserting it into the oral cavity due to trismus. It was exchanged for a McGrath® #3 blade, facilitating an approach to the epiglottis. However, the Cormack grade was evaluated as III. After applying lidocaine jelly to the bilateral nasal cavities, a cuffed tracheal tube measuring 7.0 mm in inner diameter was transnasally inserted from the left nasal cavity to the pharynx. The larynx was spread using a McGrath® #3 blade and examined by inserting a soft type of fiber optic bronchoscope (FOB) (Olympus) into the tube, facilitating visual recognition of the glottis. After spraying 8% lidocaine onto the glottis, the FOB was inserted from the subglottic area into the trachea, and FOB-guided intubation was attempted; however, difficulties were associated with inserting the tube into the subglottic area due to the stimulation of the site of the fissure and a reflex to it. Symptoms of airway obstruction appeared. Since percutaneous oxygen saturation decreased to 60%, sevoflurane (1%) was administered, followed by the intravenous administration of succinylcholine at 0.9 mg/kg, facilitating positive-pressure ventilation. While spreading the larynx again, FOB-guided intubation was attempted. Even in the state of muscle relaxation, it was difficult to insert the cuffed tracheal tube for adults, measuring 7.0 mm in inner diameter, into the trachea. A cuff-free tracheal tube measuring 6.0 mm in inner diameter was transnasally inserted into the trachea and exchanged for a cuffed tube measuring 6.5 mm in inner diameter using a soft tip-type catheter for endotracheal tube exchange (Cook Medical Corporation). The cuffed tube was fixed. The interval from the start of anesthesia until intubation was 35 min. The oral insertion of a gastric tube was then possible; however, it was impossible to insert a transesophageal echocardiographic probe. Since the cervical shape made internal jugular vein puncture impossible, a central venous catheter was inserted by 20 cm through the right femoral vein. A pulmonary artery catheter was not inserted, and an arterial pressure cardiac output monitor (Flo Trac Sensor®) (Edwards Lifesciences Corporation) was used. The surgery progressed without complications. The aortic valve was resected under cardiopulmonary bypass and substituted for a 19-mm mechanical valve. The operative time was 3 h and 5 min. The duration of cardiopulmonary bypass was 1 h and 31 min. The duration of aortic blockage was 1 h and 10 min. The volume of blood loss was approximately 2000 g. Two units of packed autologous red blood cells and 2 units of frozen fresh autologous plasma were used for blood transfusion. After surgery, artificial respiratory care was performed under continuous sedation with propofol at 0.4 to 3.0 mg/kg/h in the intensive care unit (ICU). On the first postoperative day, awakening was achieved, and the artificial respirator was removed. Laryngeal development was attempted before extubation; however, the strong pharyngeal reflex made the visual recognition of the larynx impossible. After confirming mouth opening and tongue motility, extubation was conducted while inserting a tube exchanger (Fig. 1). After extubation, there were no symptoms of upper airway obstruction or nasal hemorrhage. Since intubation was more difficult than expected, the airway was reassessed after surgery. Cervical/thoracic computed tomography (CT) was performed. The nasopharynx and larynx were subsequently examined using a transnasal fiberscope by an otorhinolaryngologist. Transnasal fiberscopy indicated adenoid vegetation, pharyngolaryngeal narrowing, and favorable glottic mobility; however, the subglottic area was difficult to observe (Fig. 2a, b). On a three-dimensional (3D) cervical CT, the airway was patent (Fig. 3) and the cervical vertebrae comprised a straight neck. The glottis was present at the level of the sixth cervical vertebra. Furthermore, displacement/circumflex of the airway axis involving the upper airway to the subglottic area was noted (Fig. 4a, b).

## Discussion

MPS type 2 is a sex-linked recessive disorder in which the abnormal intracellular lysosome deposition of a mucopolysaccharide, GAG, related to sulfoiduronate sulfatase deficiency causes progressive organ disorder. It develops in boys, with an incidence of 1/170,000 persons. Complications include airway disorder, mental retardation, a characteristic face, heart disease, and skeletal deformities. Mental retardation is observed in 75% of patients with MPS type 2, and most patients die of progressive airway/heart diseases before 20 years of age (severe MPS type 2). Mild MPS type 2 does not frequently induce mental retardation, and some patients survive until adulthood and live a social life, as demonstrated in the present case [1]. However, airway management for general anesthesia during adulthood is more difficult than during childhood due to the progression of skeletal deformities of the vertebrae/chest, deformity/stenosis of the airway, or tracheomalacia [2]. Abnormal mucopolysaccharide deposition may cause coronary artery disease, valvular disease, cardiomyopathy, vascular degeneration, or conduction disturbance. Previous studies reported that 57% of patients with MPS type 2 developed valvular disease and that the mitral valve was more frequently affected than the aortic valve. In addition, valvular regurgitation was more frequent than valvular stenosis [2, 3].

Although many studies have reported anesthetic management in children/adults with MPS type 2, few have described anesthetic management during cardiac surgery in adults with MPS type 2. When inducing anesthesia for cardiac surgery in adults with MPS type 2, it is important to consider the difficulties associated with perioperative airway management, as indicated for non-cardiac surgery in those with the same disease.

A previous study reported that airway obstruction and pulmonary edema occurred despite extubation after awakening from anesthesia in a patient with MPS type 2 for whom endotracheal intubation was performed [4]. Another study recommended that manual ventilation with a mask or respiratory care using a supraglottic instrument needs to be performed in order to avoid intubation during short-time surgery, thereby preventing airway obstruction after extubation [2]. For the present patient, endotracheal intubation was considered to be necessary for the following reasons: cardiac surgery and the necessity of postoperative artificial respiratory care.

The issue was whether awake intubation or rapid induction should be selected for a young male patient with possible airway obstruction by anesthesia/sedation. We examined airway management retrospectively referring to the guidelines for airway management prepared by the Japanese Society of Anesthesiologists [5]. Among the 12 risk factors in the “model to predict the possibility that difficulties in mask ventilation and endotracheal intubation may simultaneously occur (cannot ventilate cannot intubate; CVCI)” described in the guidelines, our patient met seven: Mallampati grade III or higher, male gender, presence of teeth, a thick neck, sleep apnea, disturbance of cervical vertebral excursion, and a short distance between the thyroid and jaw. The condition was evaluated as class V. The odds ratio for a class I (number of risk factors ≤ 3) was 18.4, and for patients in this group, there was an option of intubation under consciousness and spontaneous respiration. However, this may be difficult to apply for some patients including children, uncooperative patient, and those with a strong pharyngeal cough reflex. For the present patient, we selected tracheal intubation under spontaneous respiration and sedation. As sedation can depress or abolish the airway reflexes and can increase the risk of airway obstruction depending on its level, intubation under sedation is not recommended in the guidelines. During tracheal tube insertion, the difficulty “cannot ventilate” occurred due to reflex and neuromuscular blockade facilitated mask ventilation. This indicated that rapid induction may have been possible and safer for this patient despite the relatively higher risk of “cannot ventilate.” Furthermore, intubation should be avoided under light anesthesia or incomplete neuromuscular blockade as suggested in the guidelines. It should be noted that actual incidence of CVCI in class V group was very low (3.31%) indicating a large false-positive rate. In many patients with MPS type 2, the cervical shape makes tracheotomy difficult [6]; thus, the appropriate airway management for such patients may be rapid induction with using an extracorporeal membrane oxygenator [2] or awakening patient in case of CVCI.

Previous studies recommended the oral insertion of a supraglottic instrument and tracheal intubation through this instrument at the onset of airway obstruction [2, 5, 7]; however, these procedures were not attempted for the present patient. When trismus or the disturbance of cervical vertebral excursion is marked, intubation with an FOB is useful for visually recognizing the glottis or guiding a tube into the trachea. In some cases, a transnasal approach is more advantageous than an oral approach regarding of median scope maintenance and a laryngeal examination [8]. However, as nasal hemorrhage hinders the visual field; intranasal preparations are necessary before insertion [2]. In the present patient, the surgery required heparinization, and a tube was carefully inserted. Massive hemorrhage did not occur during surgery or after extubation.

We were unable to predict subglottic stenosis/deformity or difficulties with tracheal tube passage based on preoperative coronal MRI sections. In the postoperative airway assessment, the FOB was useful for evaluating narrowing related to oropharyngeal soft tissue outgrowth or subglottic stenosis. On the other hand, neither findings were recognized in the CT-/MRI-based image assessment. Based on 3D CT images of the airway, it was possible to evaluate the morphology of the airway or displacement/circumflex of the airway axis, whereas the soft tissue assessment was limited; more issues remain to be resolved than those associated with an airway assessment with an FOB [9].

## Conclusions

We encountered a patient with adult MPS type 2 for whom airway management for aortic valve replacement was difficult. Multiple factors contributed to this difficulty, and airway management strategies were restricted due to the characteristics of this disease, especially tracheostomy could be extremely difficult. Either awake intubation or rapid induction can be selected for this patient; however, either way have risks of airway obstruction. It is important that strategies under light anesthesia or incomplete neuromuscular blockade should be avoided as suggested in the guidelines. Thus, a preoperative multidisciplinary airway assessment and simulation are important.

## Abbreviations

3D: Three-dimensional
CT: Computed tomography
CVCI: Cannot ventilate cannot intubate
FOB: Fiber optic bronchoscope
GAG: Glycosaminoglycan
ICU: Intensive care unit
MPS: Mucopolysaccharidosis
MRI: Magnetic resonance imaging
RASS: Richmond Agitation Scale Score
